# Novel emerging reassortant H6 avian influenza viruses with internal genes from G57 genotype of H9N2 pose potential zoonotic risk

**DOI:** 10.1080/22221751.2026.2732846

**Published:** 2026-09-18

**Authors:** Zhimin Wan, Wenjie Jiang, Xudong Cao, Xingyao Guo, Xinyan He, Jianjun Zhang, Xinran Chu, Xinyi Ji, Yu Liu, Xuefeng Yin, Zhehong Zhao, Jixiang Wang, Wei Gao, Quan Xie, Tuofan Li, Hongxia Shao, Aijian Qin, Yuhai Bi, Jianqiang Ye

**Affiliations:** a Key Laboratory of Jiangsu Preventive Veterinary Medicine, Key Laboratory for Avian Preventive Medicine, Ministry of Education, College of Veterinary Medicine, Yangzhou University, Yangzhou, People’s Republic of China; b Jiangsu Co-innovation Center for Prevention and Control of Important Animal Infectious Diseases and Zoonoses, Yangzhou University, Yangzhou, People’s Republic of China; c Joint International Research Laboratory of Agriculture and Agri-Product Safety, the Ministry of Education of China, Yangzhou University, Yangzhou, People’s Republic of China; d Jiangsu Key Laboratory of Zoonosis, Yangzhou University, Yangzhou, People’s Republic of China; e Sinopharm Yangzhou VAC Biological Engineering Co. Ltd., Yangzhou, People’s Republic of China; f Institute of Microbiology, Chinese Academy of Sciences, Beijing, People’s Republic of China; g University of Chinese Academy of Sciences, Beijing, People’s Republic of China; h Beijing Research Center for Respiratory Infectious Diseases, Beijing Key Laboratory of Surveillance, Early Warning and Pathogen Research on Emerging Infectious Diseases, Beijing, People’s Republic of China

**Keywords:** H6 AIV, reassortment, receptor binding, replication, pathogenicity, mouse, chicken

## Abstract

In recent decades, multiple novel reassortant avian influenza viruses (AIVs) carrying internal genes derived from H9N2 viruses have emerged in poultry in China and have repeatedly caused human infections, highlighting the pivotal role of the H9N2 internal genes cassette in facilitating cross-species transmission. In this study, five H6N1 and one H6N2 AIVs were isolated from chickens and Houdan chickens exhibiting respiratory symptoms. Genetic analyses revealed that the heamagglutinin (HA) genes of these H6 AIVs belonged to the ST339-like lineage and originated from H6 AIVs circulating in domestic geese in China, whereas the neuraminidase (NA) genes were derived from H9N2 or H5N1 AIVs, respectively. Notably, all six internal genes of these H6 isolates were derived from the G57 genotype H9N2 virus. *In vitro* studies demonstrated that these H6 AIVs replicated effectively in both avian and mammalian cells. The H6N2 isolate displayed dual receptor binding affinity for both avian-like and human-like receptors, whereas the five H6N1 viruses showed no detectable receptor binding affinity under the assay conditions *in vitro*. Reverse-genetics reassortant assay further revealed that the H6N1 HA itself possesses intrinsic dual receptor binding affinity. Furthermore, these H6 isolates exhibited high virulence in mice, and induced obvious respiratory symptoms in chickens, with efficient transmission among birds. Collectively, our findings demonstrate that H6 AIVs carrying the G57 H9N2 internal gene constellation are actively circulating in chicken populations and pose a substantial threat to poultry health, underscoring their potential risk to public health and the need for continued surveillance at the poultry-human interface.

## Introduction

Avian influenza virus (AIV), a member of the *Orthomyxoviridae* family, possesses a segmented genome consisting of eight negative-sense, single-stranded RNAs that encode 8 structural proteins and several non-structural proteins. Based on the antigenic properties of two surface glycoproteins, hemagglutinin (HA) and neuraminidase (NA), influenza A viruses (IAVs) are classified into 18 HA and 11 NA subtypes [1]. With the exception of H17N10 and H18N11, which were detected in bats, all HA and NA subtypes have been identified in wild birds, particularly migratory waterfowl, which serve as the primary natural reservoir of AIVs [2]. Recently, a novel candidate HA subtype, designated H19, was identified in wild birds [3]. AIVs not only cause substantial economic losses to the poultry industry but also pose a serious threat to public health due to their ability to cross species barriers and infect humans.

In recent decades, several AIV subtypes, including H7N9, H5Ny, H10N3, H10N8 and H3N8, have been prevalent among chicken flocks in China and have repeatedly infected humans [4–8]. Notably, these AIVs share internal genes derived from H9N2 AIVs, the most prevalent subtype of AIVs in China [9]. These findings raise the possibility that the internal genes of H9N2 viruses may act as a genetic backbone driving the emergence of novel reassortant AIVs with enhanced fitness in chickens and increased zoonotic or even pandemic potential. The H6 subtype AIV was first isolated from turkeys in United States in 1965 [10]. Since then, H6 viruses have been widely distributed across domestic poultry and wild birds in Eurasia and North America [11], with increasing genomic diversity driven by frequent reassortment and long-range bird migration [12–15]. National wild surveillance revealed extensive circulation and substantial genotypic variation of H6 AIVs in multiple hosts in China [16, 17]. H6 AIVs are typically classified as low pathogenic AIVs (LPAIVs) and are among the most commonly isolated subtypes in live-bird markets, wild birds and poultry flocks in China and Southeast Asia, including H6N1, H6N2, H6N5, H6N6 and H6N8 isolates with demonstrated intercontinental lineage mixing [18, 19]. Importantly, H6 AIVs have demonstrated the capacity to infect mammals, as evidenced by efficient replication in mice without prior adaptation [20, 21], a shift towards mammalian-like receptor-binding preferences [22], natural spill-over events in pigs [23, 24], and a documented human H6N1 infection in Taiwan [25], underscoring their zoonotic potential.

Although frequent introductions of H6 AIVs from wild birds into domestic poultry have been documented, historically the onward transmission was limited [26]. However, in late 2025, outbreaks of H6 AIVs were reported in chicken flocks in China, causing respiratory disease characterized by bronchial obstruction. In this study, we identified and characterized novel H6N1 and H6N2 AIVs isolated from chickens, including Houdan chickens exhibiting respiratory symptoms. Genetic analyses revealed that these viruses are reassortants comprising HA genes derived from goose-origin H6 viruses, NA genes from wild bird- or duck-origin H5N1 (N1) viruses, and internal gene segments and/or N2 genes originating from the G57 genotype H9N2 viruses. These H6 AIVs replicated efficiently in both avian and mammalian cells. Furthermore, *in vivo* experiments demonstrated high virulence in mice and efficient transmission among chickens, highlighting their potential threat to public health and emphasizing the urgent need for enhanced surveillance and risk assessment.

## Materials and methods

### Viral isolation and identification

Throat swab samples were collected from chickens and Houdan chickens presenting respiratory symptoms in China during 2025. The samples were inoculated into 10-day-old specific-pathogen-free (SPF) embryonated chicken eggs and incubated for 72 h . Allantoic fluid was harvested and screened for hemagglutination activity following standard protocols. Viral RNA was extracted from HA-positive allantoic fluid using a commercial RNA extraction kit, and cDNA was synthesized by reverse transcription (RT) with Unit 12 primers. Eight gene segments of AIVs were amplified by PCR with the gene segment-specific primers [27], and all PCR products were sequenced by Sanger sequencing for full-genome characterization.

### Phylogenetic analysis

The genomic sequences of additional AIVs were obtained from the previous epidemiological surveillance conducted in our laboratory, as well as from public databases of the Global Initiative on Sharing Avian Influenza Data (GISAID) and National Center for Biotechnology Information (NCBI). Multiple sequences were aligned by the MAFFT (v7.530). Bayesian evolutionary analyses of HA and NA genes were performed in BEAST (v2.7.8). The general time-reversible plus gamma distribution with four rate categories and invariable sites (GTR + G4 + I) model was used for nucleotide substitution. An uncorrelated lognormal relaxed molecular clock was applied to account for rate heterogeneity among lineages. The Bayesian Skyline Plot (BSP) was chosen as the tree prior to infer changes in effective population size over time. Markov Chain Monte Carlo (MCMC) simulations were run for 10,000,000 generations, with automatic sampling frequency to ensure adequate convergence. ThreeAnnotator (v2.7.7) was used to summarize the posterior distribution of the trees generated by MCMC. After discarding the first 10% of samples as burn-in, a maximum clade credibility (MCC) tree was constructed, and posterior probabilities of each node were calculated as branch support values. The resulting phylogenetic trees were visualized and edited using iTOL and FigTree (v1.4.4). Phylogenetic trees of internal genes were constructed using the neighbor-joining method with bootstrap analysis (1,000 replicates) in MEGA 11 and visualized with iTOL.

### Hemagglutination elution assay

The H6 isolates were 2-fold serially diluted in 96-well plates and incubated with different species RBCs for 1 h at 4°C to allow hemagglutination. Then the plates were transferred to 37°C to initiate elution. The HA titres of each virus were recorded every 0.5 h until complete elution was observed.

### NA activity assay

NA activity of the H6 isolates was measured using a fluorometric assay with 4-(methylumbelliferyl)-N-acetylneuraminic acid (MUNANA, Sigma-Aldrich, USA) as the substrate. Briefly, all H6 isolates were standardized to 2^6^ hemagglutination units (HAU) and subjected to 2-fold serial dilution (50 μL/well) in 96-well black microplate (Thermo Fisher, USA). Subsequently, 50 μL of 100 μmol/L MUNANA was added to each well, and the mixture was incubated at 37°C for 1 h. The reaction was terminated with 100 μL of 0.2 mol/L Na_2_CO_3_. Fluorescence intensity was detected using a BioTek Synergy-2 microplate reader (excitation at 360 nm and emission at 460 nm). The NA activity was expressed as relative fluorescence units (PRU), which were calculated by subtracting the mean value of the negative control from the raw reading.

### Titration of 50% tissue culture infectious dose (TCID_50_)

Ninety per cent confluent chicken embryo fibroblasts (CEF), Madin-Darby Canine kidney (MDCK) cells, and human lung adenocarcinoma A549 cells were infected with 10-fold serial dilutions of H6 viruses and incubated for 2 h. Then, the supernatants were removed, and the infected cells were washed two times with PBS and maintained in opti-MEM containing 1 μg/mL TPCK-trypsin. After 72 h, the supernatants were tested by HA assay. The TCID_50_ value was calculated by the method of Reed and Muench.

### Viral growth kinetics

CEF, MDCK and A549 cells were seeded to form confluent monolayers in 6-well plates and infected with H6 isolates at a multiplicity of infection (MOI) of 0.01. Infected cells were maintained in opti-MEM medium supplemented with 1 μg/mL TPCK-trypsin and incubated at 37°C. Cell supernatants were collected at 6, 12, 24, 48, and 72 h post-infection (hpi). Viral titres in supernatants were determined by TCID_50_ and calculated by the Reed-Muench method. The kinetic assays were performed in triplicate.

### Viral plaque assay

The plaque phenotype of the H6 isolates was analyzed in MDCK cells using 1% agar overlay. Briefly, MDCK cells in 6-well plates were infected with 10-fold serial dilutions of each virus. After adsorption for 2 h, the supernatants were removed, the infected cells were overlaid with 1% agar containing TPCK-trypsin and incubated for 72 h at 37°C and 5% CO_2_. The cells were fixed with 4% paraformaldehyde, and then stained with 0.1% crystal violet solution.

### Plasmid construction and virus rescue

The HA, PB2, PB1, PA and NP of some H6 strains were cloned into the plasmid pDP2002 according to a previous report. The segments and the linearized influenza vector pDP2002 were amplified. Each segment of H6 strains was recombined with the linearized pDP2002 vector using the Exnase^TM^ II according to the manufacturer’s instructions. The constructed plasmids were sequenced by Sanger sequencing.

The H6 isolate was rescued with the A/chicken/Xuzhou/508/2019(H9N2) (XZ508) backbone by reverse genetics as previously described [28]. Briefly, eight plasmids (HA of H6N1 isolate CZ56 and the remaining seven plasmids from XZ508) were co-transfected into co-cultured 293 T and MDCK cells. After 6 h of incubation, the culture medium was replaced with 1 mL of fresh opti-MEM. After 18 h of incubation, the medium was exchanged with 2 mL of fresh opti-MEM medium containing 1 μg/mL TPCK-Trypsin. At 3 d post-transfection, the supernatants were collected and confirmed by HA assay, followed by inoculation into SPF embryonated chicken eggs for virus amplification.

### Receptor binding preference analysis

The receptor binding specificity of H6 isolates was analyzed as previously described, using two different glycopolymers: Neu5Acα2-3Galb1-4GlcNAcb-sp3-PAA-biotin (avian-like receptor) and Neu5Acα2-6Galb1-4GlcNAcb-sp3-PAA-biotin (human-like receptor) (Glycitech, USA) [29]. Briefly, the two glycopolymers were diluted to 5, 0.2, and 0.008 μg/mL and coated onto the Pierce streptavidin high binding capacity coated 96-well plates (100 μL/well). Plates were blocked with 5% skim milk in PBST, then 64 HAUs of the H6 isolates were added and incubated at 4°C overnight. After three washes, the plates were incubated with H6-specific monoclonal antibody (MAb) 4C2, which was generated in our laboratory [30]. The plates were washed three times with PBST and incubated with HRP-conjugated anti-mouse IgG antibody for 2 h and then washed 3 times with PBST. Colour development was performed with TMB substrate (100 μL/well). The reaction was stopped by adding 2 M H_2_SO_4_ and the OD450 were measured.

### Mouse challenge study

Five-week-old BALB/c mice were used to assess the pathogenicity of the H6 isolates. All mice were randomly assigned to three groups (11 mice/group) and anesthetized with isoflurane, with all mice in each group being intranasally inoculated with 10^5^ TCID_50_ of each virus in 25 μL volume. Body weight and survival rates were monitored over a 14-day period. At 3- and 6-days post infection (dpi), three mice in each group were sacrificed, and nasal turbinate and lung samples were collected to determine viral titres. Additionally, lung samples collected at 5 dpi were also used for histopathology analysis. The mice with body weight loss exceeding 25% will be anesthetized and euthanized.

### Chicken challenge study

Ten-day-old SPF chickens were randomly distributed into different challenge groups (10^6^ chickens/group) and infected intranasally with H6 isolate in 0.1 mL. Four uninfected SPF chickens were introduced into each challenged group as a direct-contact group. At 3 and 5 dpi, 3 chickens per each challenged group were humanely euthanized, and the trachea and lung tissues were harvested for viral titration. Oropharyngeal and cloaca swabs were collected at 2, 4, 6, 8, and 10 dpi to monitor viral shedding.

### Hemagglutination inhibition (HI) assay

The H6 antisera were initially subjected to 10-fold primary dilutions, followed by serial dilutions in 96-well plates and mixed with 8 hemagglutination units (HAU) of virus. After incubation at room temperature (RT) for 30 min, 0.5% red blood cells were added to each well and incubated at RT for 30 min before the HI titres were determined.

### Luciferase assay for polymerase activity

Polymerase activity was measured using mini-genome assay as previously described [31]. Briefly, 293 T cells were co-transfected with a luciferase reporter plasmid, p-Luci, together with RNP plasmids from H6 isolates and an internal control Renilla plasmid. After 48 h, the luciferase activity was measure using a dual-luciferase reporter system. The polymerase activity assays were performed in triplicate.

### Ethics statement and biosafety

All animal studies were conducted in strict accordance with the institutional animal guidelines approved by the Animal Care Committee at Yangzhou University, China. The protocols were approved by the Animal Committee of Yangzhou University with ethical permission numbers 202402035 and 202402137. All operations involving influenza viruses were performed in biosafety level 2 (BSL-2) and animal biosafety level 2 (ABSL-2) facilities.

### Statistical analysis

The statistical analysis in the study was performed with two-way ANOVA using GraphPad 8. A *p* value of less than 0.05 was considered significant. *, **, ***, and **** indicate *p* values of less than 0.05, 0.01, 0.001 and 0.0001, respectively.

## Results

### Isolation and identification of novel reassortant H6 viruses

To isolate viruses, throat swabs from chickens and Houdan chickens with respiratory symptoms were collected and inoculated into 10-day-old SPF embryonated chicken eggs. After incubation, the allantoic fluids were harvested and screened for AIV by HA assay and RT-PCR. The positive samples were subjected to whole-genome sequencing. The viruses were purified by plaque assay, and six H6 isolates were finally obtained, designated as A/chicken/Changzhou/56/2025(H6N1) (CZ56), A/chicken/Changzhou/57/2025(H6N1) (CZ57), A/chicken/Changzhou/58/2025(H6N1) (CZ58), A/chicken/Changzhou/511/2025(H6N1) (CZ511), A/chicken/Anhui/25107/2025(H6N1) (AH25107), and A/Houdan chicken/Nanjing/2501/2025(H6N2) (NJ2501). All the sequences were submitted to the GISAID (accession no: EPI_ISL_20504196 and EPI_ISL_20504395–EPI_ISL_20504399).

**Figure 1.**
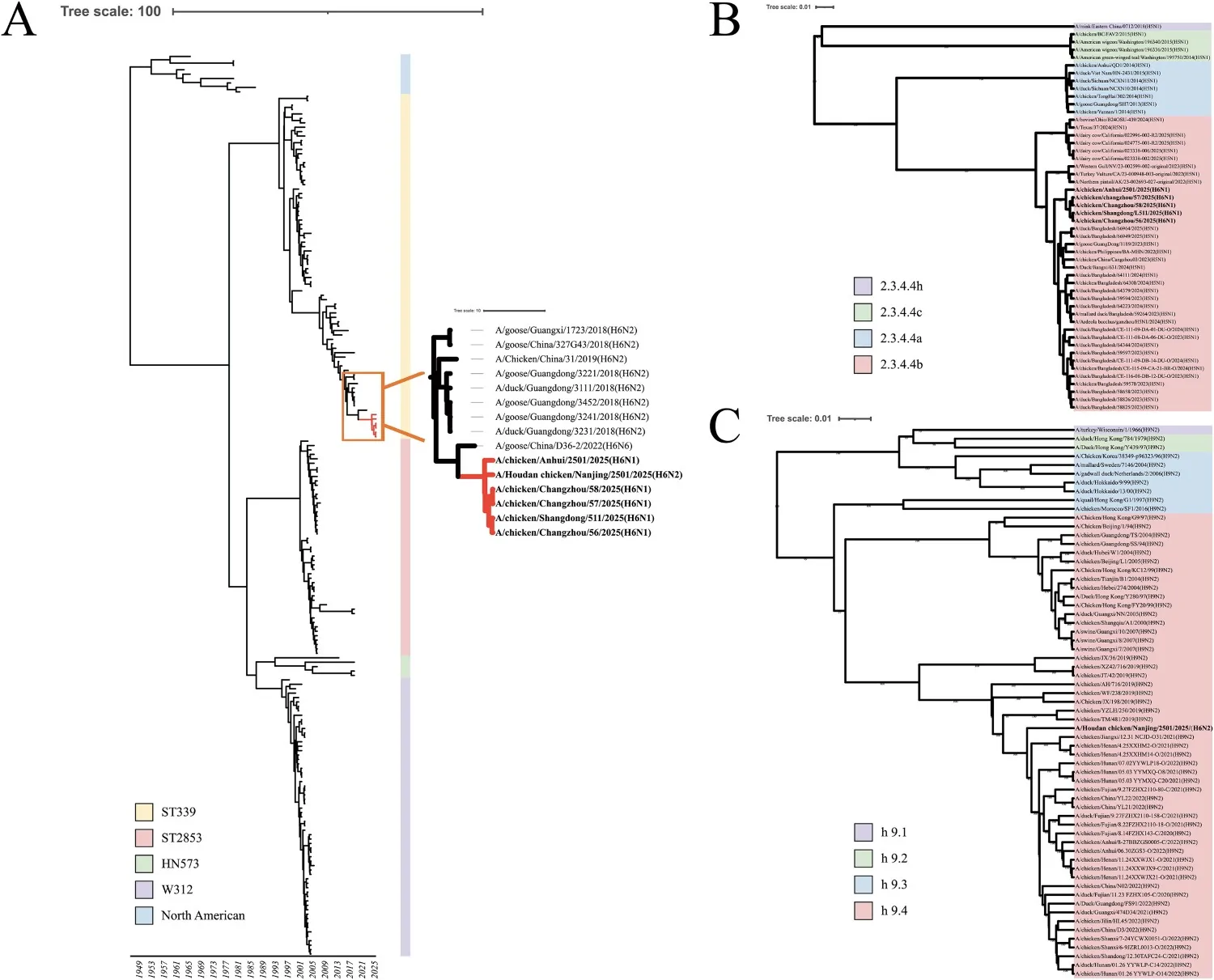
Phylogenetic analyses of surface protein genes (A) H6, (B) N1 and (C) N2 of the six H6 AIVs isolated from chickens in China. The MCC trees of HA and NA were analyzed using TreeAnnotator with common ancestor heights following the burn-in of the first 10%. The phylogenetic trees were visualized and edited using iTOL and FigTree.

**Figure 2.**
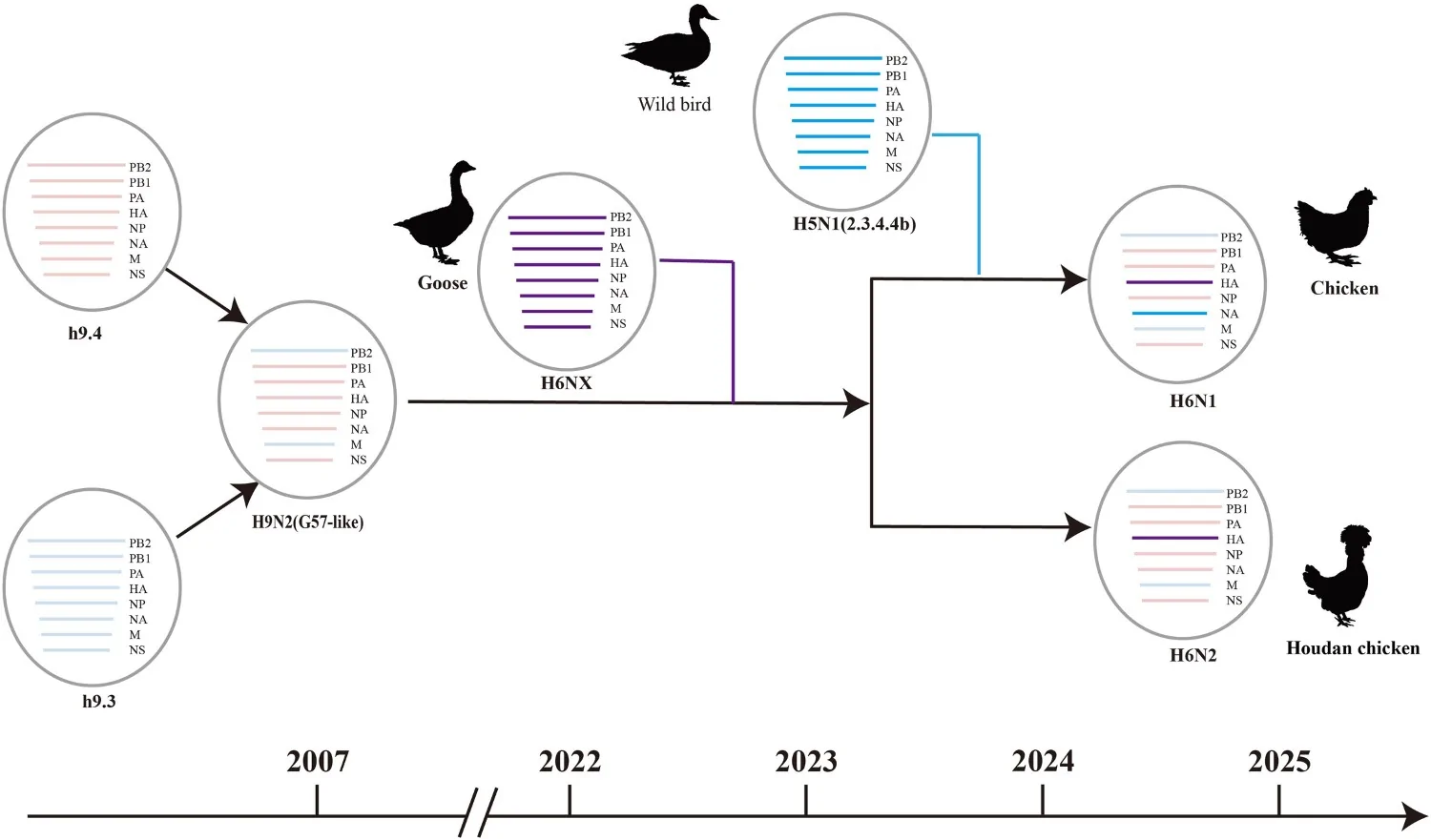
Schematic representation of the inferred reassortment history of the six H6 AIVs isolated from chickens in China. Origins of the eight segments and their putative donor hosts are shown. The genes of h9.3-like and h9.4-like H9N2, goose-origin H6, and 2.3.4.4b H5N1 virus are marked light-blue, pink, purple, and bright-blue, respectively.

To trace the evolutionary origins of these H6 isolates, phylogenetic analyses of the HA and NA genes were performed. As shown in Figure 1(A), all HA genes of the six H6 isolates belonged to the ST339-like lineage, and originated from H6N2 AIVs circulating in domestic geese in China. The NA gene of the H6N2 isolate (NJ2501) was closely related to H9N2 viruses circulating in chicken flocks in China (Figure 1(B)), whereas the NA gene of five H6N1 isolates fell into a sub-lineage derived from H5N1 viruses circulating in ducks and wild birds in Southeast Asia (Figure 1(C)). Analysis of the six internal genes revealed a uniform H9N2 backbone. Among these six internal genes, the PB2 and M genes belonged to h9.4 (G1-like), while the other four genes (PB1, PA, NP and NS) were assigned to the h9.3 (BJ/94-like) lineage H9N2 viruses (Supplementary Figure 1). These results indicate that the internal genes of the six H6 isolates belonged to the G57 genotype of H9N2 virus, which is the prevalent H9N2 strain in chicken flocks in China. Taken together, these data show that the H6 isolates are novel reassortant viruses: HA derived from goose-origin H6 virus, NA acquired from H9N2 (chicken) or H5N1 (duck/wild bird) pools, and internal genes were from G57 genotype H9N2 viruses. To intuitively visualize the multi-source reassortment pattern of these H6 isolates, we generated a schematic diagram (Figure 2) summarizing the origin of each of the eight genomic segments, putative donor host species, and the inferred reassortment timeline.

### Molecular characterization of the novel H6 isolates

To evaluate the potential risk of these novel H6 isolates spilling over to mammals, the key amino acids of these viruses associated with pathogenicity and transmission were analyzed. The amino acid sequence at the HA cleavage site of all the six H6 isolates was PQIETR/GLF, indicating these H6 isolates belonged to low pathogenic AIV (LPAIV). The critical mutations in the HA receptor binding site that mediate a switch from avian-type to human-type, e.g. Q226L/R and G228S, were not found in these novel H6 isolates (Table 1). Notably, although E627 K [32, 33] and D701N [34] in PB2 for pathogenesis in mammals and H276Y in NA (N2 numbering) [35, 36] for reducing susceptibility to oseltamivir were not found, some mutations associated with increased polymerase activity and enhanced pathogenicity in mice were detected in some or all isolates (Table 1). Such as A588 V, V598I, S75N in PB2, 3 V, 622G in PB1, V63I, P190S, K356R, 383D, D400P, N409S, and 615N in PA, N42S, 138F, 149A in NP, N30D, I43M, and T215A [37]. Furthermore, V27A and S31N of M2, which can increase resistance to amantadine and rimantadine of AIV [37], were present in part or all H6 isolates.

**Table 1.** Molecular characterization of H6 AIVs.

Protein	Genetic marker	H6 isolates						Function
		NJ2501	CZ56	CZ57	CZ58	CZ511	AH25107	
PB2	A588V	V	V	V	V	V	V	Increased polymerase activity and virulence in mice
	V598I	I	I	I	I	I	I	
	E627K	E	E	E	E	E	E	
	D701N	D	D	D	D	D	D	
PB1	D3V	V	V	V	V	V	V	Increase polymerase activity in avian and mammalian cells
	D622G	G	G	G	G	G	G	Increase polymerase activity and virulence in mice
PA	V63I	V	V	I	I	V	I	Increase polymerase activity and virulence in mice
	K356R	R	K	R	R	K	R	
	N383D	D	D	D	D	D	D	Increase polymerase activity in mammalian and avian cells
	N409S	N	S	N	N	S	N	Increase polymerase activity in mammalian cells
HA^a^	Q226L/R	Q	Q	Q	Q	Q	Q	Increase binding to human-like receptor
	G228S	G	G	G	G	G	G	
NA^b^	H274Y	H	H	H	H	H	H	Reduce susceptibility to oseltamivir
M1	N30D	D	D	D	D	D	D	Increase virulence in mice
	I43M	M	M	M	M	M	M	
	T215A	A	A	A	A	A	A	
M2	V27A	A	V	V	V	V	A	Increase resistance to amantadine and rimantadine
	S31N	N	N	N	N	N	N	
NS1	C138F	F	F	F	F	F	F	Increase replication in mammalian cells and virulence in mice

^a^ H3 number.

^b^ N2 number.

### The hemagglutination and elution activity of the H6 isolates

**Figure 3.**
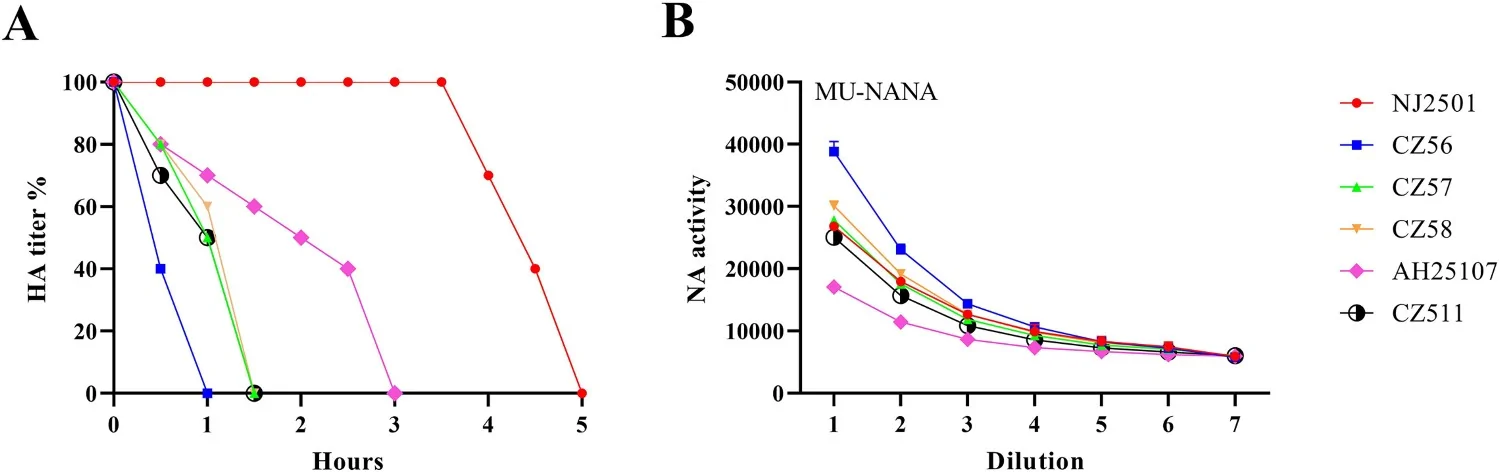
Elution kinetics and NA activity of the H6 isolates. (A) Elution kinetics of the H6 isolates. The H6 isolates were serially 2-fold diluted and incubated with chicken RBCs for 1 h at 4°C. Then the plates were transferred to 37°C to initiate elution. The HA titres were recorded every 0.5 h until complete elution was observed, and the elution kinetics were analyzed accordingly. (B) The NA activity of the H6 isolates. The serially 2-fold diluted H6 isolates were mixed with MUNANA and incubated 1 h at 37°C. Fluorescence intensity was detected upon excitation at 360 nm and emission at 460 nm.

To evaluate the hemagglutination ability of the H6 isolates, hemagglutination assays were performed using chicken, goose, turkey, and guinea pig RBCs. All the H6 isolates exhibited high HA titres (HA titres ≥1:1024), with notably higher titres observed with turkey and guinea pig RBCs (Table 2), suggesting a broad receptor-binding ability. To further characterize viral release properties, we performed the hemagglutination elution assays using chicken RBCs. Clear different elution kinetics were observed among the six H6 strains. NJ2501 exhibited the slowest elution, requiring approximately 5 h for complete elution, while AH25107 required 3 h (Figure 3(A)). In contrast, CZ57, CZ58, and CZ511 eluted more rapidly (2 h), and CZ56 displayed the fastest elution among all test strains, completing within 1 h (Figure 3(A)). Given that NA activity of influenza viruses is a critical determinant of viral release from host cells, we further measured the NA enzymatic activity of these H6 isolates using MUNANA as a substrate. The results revealed that CZ56 displayed the highest NA activity, whereas AH25107 showed the lowest NA activity (Figure 3(B)). Notably, the variation in elution kinetics among the H6 isolates was generally consistent with their respective NA activities. Collectively, these findings indicate that although all H6 isolates demonstrate strong hemagglutination capacity across multiple erythrocyte species, significant variation exists in their elution kinetics, which suggests a functional balance between HA binding affinity and NA enzymatic activity.

**Table 2.** HA titres of the six H6 isolates with different species RBCs.

Virus	HA titres (Log_2_)			
	cRBC^a^	gRBC	tRBC	gpRBC
NJ2501	1:1024	1:1024	1:1024	1:4096
CZ56	1:1024	1:1024	1:8192	1:2048
CZ57	1:2048	1:1024	1:2048	1:2048
CZ58	1:2048	1:1024	1:2048	1:4096
CZ511	1:1024	1:1024	1:4096	1:4096
H25107	1:2048	1:4096	1:4096	1:4096

^a^ cRBC, gRBC, tRBC, and gpRBC represent chicken, goose, turkey, and guinea-pig red blood cells, respectively.

### Receptor-binding preference of the novel H6 isolates

**Figure 4.**
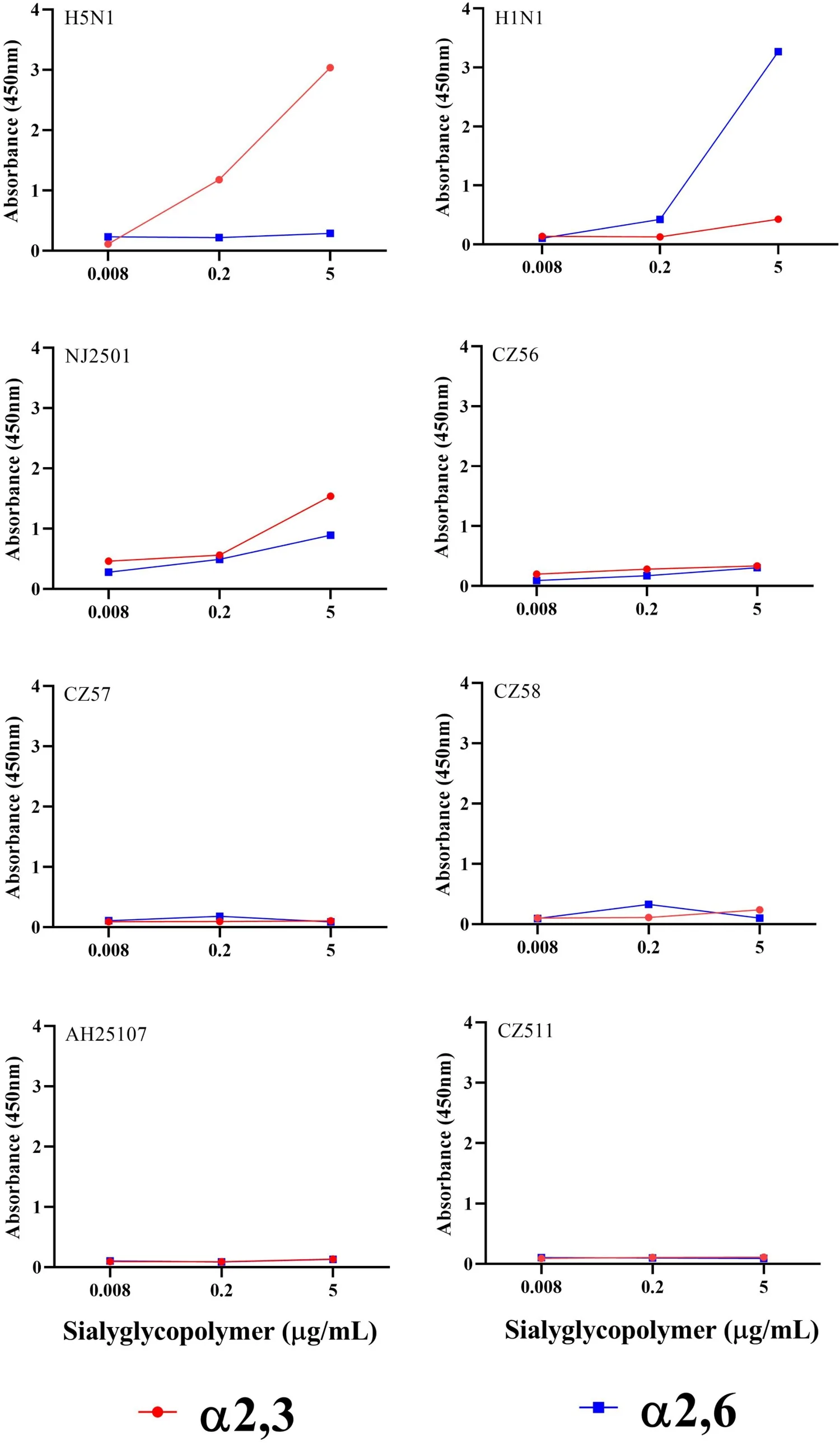
Receptor-binding properties of the H6 isolates. Receptor-binding profiles of the H6 isolates were evaluated by direct solid-phase binding assays using various concentrations of sialic acids conjugated to biotinylated sialylglycopolymers (3’SLN and 6’SLN).

To investigate the receptor binding specificity of these six H6 isolates, avian-like receptor Neu5A cα2-3Galb1-4GlcNAcb-PAA-biotin and human-like receptor Neu5Acα2-6Galb1-4GlcNAcb-PAA-biotin were used to test their receptor binding in ELISA. As described in Figure 4, H6N2 isolate NJ2501 displayed dual receptor binding affinity for both avian-like and human-like receptors, whereas the five H6N1 isolates (CZ56, CZ57, CZ58, CZ511, AH25107) showed undetectable receptor binding affinity for either avian-like or human-like receptors (Figure 4). As controls, H5N1 avian influenza virus only bound to avian-like receptor and H1N1 human influenza virus only bound to human-like receptor. Notably, although the H6N2 isolate NJ2501 had dual receptor binding activity, its binding affinity to avian-like receptor or human-like receptor was lower than that of H5N1 or H1N1, respectively. Due to the distinct receptor-binding phenotypes observed between H6N2 and H6N1, we hypothesized that the undetectable signal might be influenced by the NA background rather than by an intrinsic lack of receptor binding of the H6N1 HA protein. To investigate this possibility, we generated reassortant virus rCZ56 on the G57 genotype H9N2 backbone (XZ508) [28], which had the HA gene from CZ56 and other seven genes from XZ508. The results showed that rCZ56 displayed obvious dual receptor-binding affinity for both avian-like and human-like receptors (Supplementary Figure 2). These results demonstrate that the XZ56 HA possesses intrinsic dual receptor-binding activity. Collectively, these results demonstrate that these novel H6 isolates show binding affinity for both avian-like and human-like receptors *in vitro*.

### Growth kinetics of the novel H6 isolates in different cells

**Figure 5.**
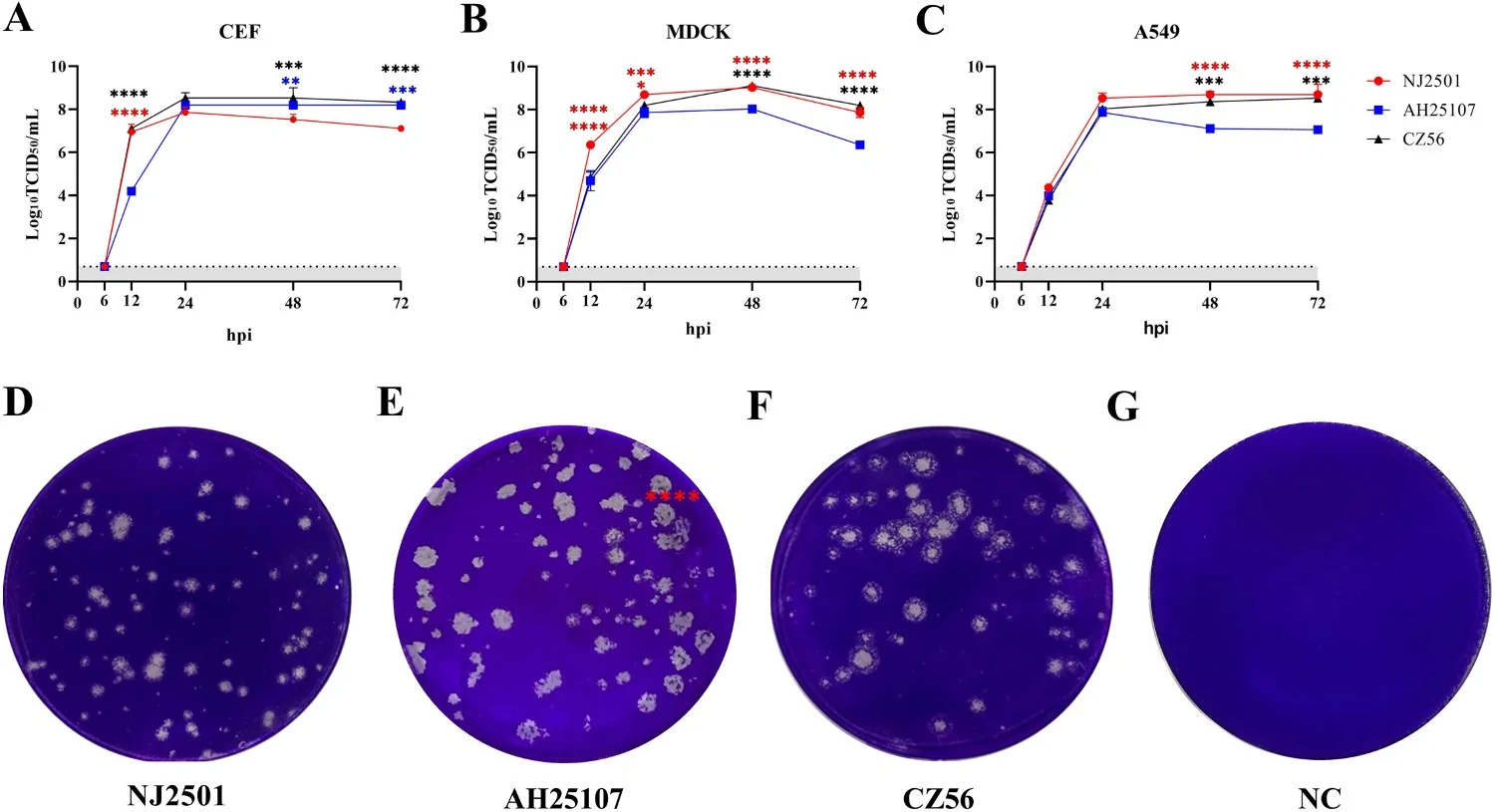
Growth kinetics and plaque phenotype of the H6 AIVs. Growth kinetics were determined in (A) CEF, (B) MDCK and (C) A549 cells. Confluent monolayers were infected with the H6 isolates at an MOI of 0.01. The supernatant samples were collected at 6, 12, 24, 48 and 72 hpi, and viral titres were determined in MDCK cells. The kinetic assays were performed in triplicate. The plaque phenotypes of (D) NJ2501, (E) AH25107 and (F) CZ56 were assessed by plaque assay on MDCK cells under a 1% agarose overlay, and (G) is a negative control (NC).

Because the H6N1 viruses (CZ56, CZ57, CZ58 and CZ511) were isolated from the same location and exhibited similar genetic characteristics (Figure 1, Supplementary Figures 1 and 2), CZ56 was selected as the representative isolate, and together with NJ2501 and AH25107, was used in the subsequent experiments. To evaluate the viral replication ability of these novel H6 isolates in avian and mammalian cells, the growth kinetics of the three selected H6 viruses in CEF, MDCK and A549 cells were examined. As shown in Figure 5, all three H6 viruses replicated effectively in both avian and mammalian cells, achieving peak titres at 24 hpi. In A549 and MDCK cells, NJ2501 and CZ56 displayed comparable replication efficiency, with peak titres reaching about 10^8^ TCID_50_/mL. Compared to CZ56, NJ2501 and AH25107 demonstrated higher replication efficiency in CEF cells between 24 and 72 hpi. To further characterize the *in vitro* replication properties of the three H6 isolates, plaque formation assays were performed in MDCK cells. As shown in Figure 5, the three H6 isolates formed clear plaques with comparable sizes. All these clearly demonstrate that these novel H6 isolates can efficiently replicate in both avian and mammalian cells without adaptation, indicating the risk of the cross-transmission of these H6 from poultry to mammalian species.

### Pathogenicity of the novel H6 isolates in mice

**Figure 6.**
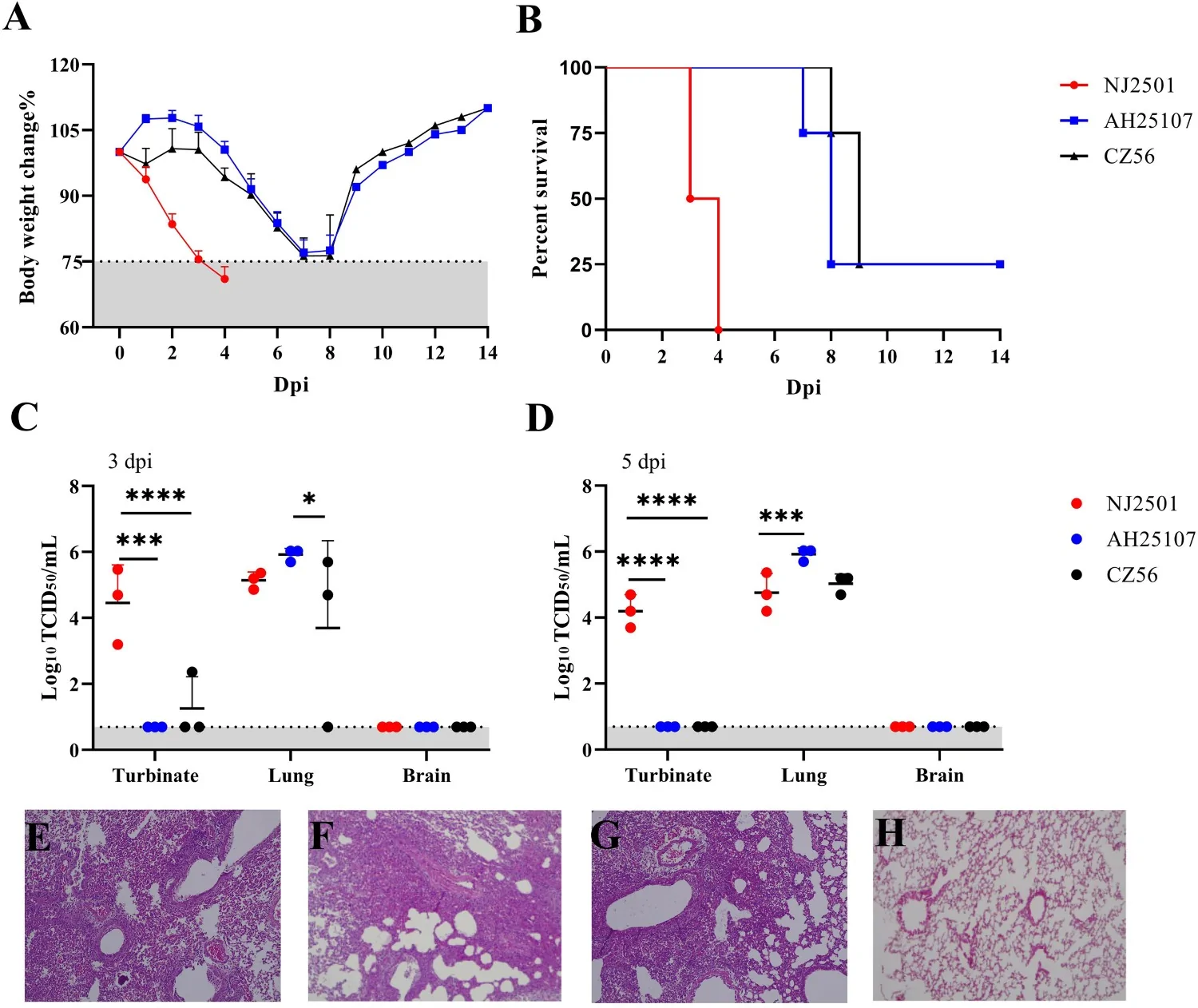
Pathogenicity of the H6 isolates in mice. Five-week-old BALB/c mice were intranasally infected with 10^5^ TCID_50_ of each H6 isolate. (A) Body weight changes and (B) survival were monitored daily for 14 days. At 3 and 5 dpi, three mice from each group were euthanized, and nasal turbinate, lung and brain samples were collected for virus titration at (C) 3 and (D) 5 dpi. Lung tissues were fixed in 10% neutral-buffered formalin, embedded in paraffin, sectioned, and stained with heamatoxylin and eosin (H&E). (E) NJ2501, (F) AH25107 and (G) CZ56 and (H) Mock.

To assess the pathogenicity of the H6 isolates in mammals, NJ2501, AH25107 and CZ56 were subjected to mouse challenge study. Five-week-old BALB/c mice were intranasally infected with 10^5^ TCID_50_ of each of the three H6 viruses. Mice infected with NJ2501 displayed obvious clinical symptoms from 2 dpi, including fur ruffling and weight loss, and rapidly succumbed to the viral infection, resulting in 100% mortality by 4 dpi (Figure 6(A, B)). Mice infected with AH25107 and CZ56 began to show significant weight loss from 4 dpi and caused 75% mortality, after which the surviving mice began to recover from 8 dpi (Figure 6(A, B)). At 3 and 5 dpi, three mice from each group were euthanized, and nasal turbinate, lung and brain samples were collected for viral titration. High viral titres were detected in the turbinate and lung samples of mice infected with NJ2501 at both 3- and 5 dpi (Figure 6(C, D)). Mice infected with AH25107 and CZ56 exhibited similar viral titres in the lungs compared with those in NJ2501 group, whereas only one mouse infected with CZ56 showed low level viral replication in the turbinate at 3 dpi, and no virus was detected in the remaining infected mice (Figure 6(C, D)). Notably, no virus was detected in brain tissues of any infected mice throughout the experiment. In addition, the lung histopathology analysis revealed that NJ2501, AH25107 and CZ56 induced severe pneumonia, characterized by the infiltration of inflammatory cells and heamorrhage (Figure 6(E–H)). These data demonstrate that H6 isolates NJ2501, AH25107 and CZ56 without adaptation are lethal to BALB/c mice.

### Polymerase activity of the viral RNP complex

**Figure 7.**
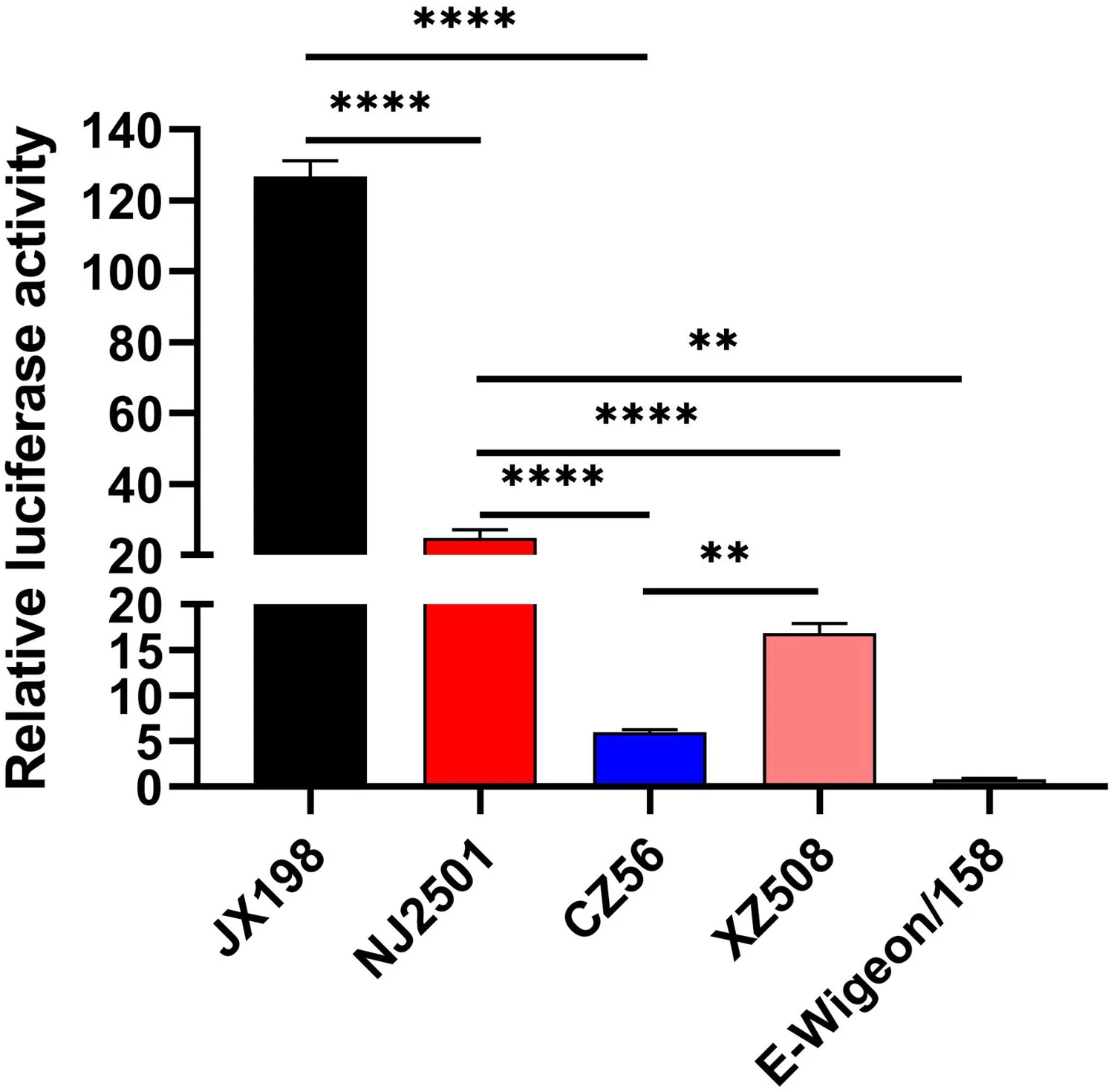
Polymerase activity of NJ2501 and CZ56 isolates. Relative RNP polymerase activity determined by mini-genome dual-luciferase reporter assay. JX198 (G57 genotype H9N2, high pathogenic in mice), XZ508 (G57 genotype H9N2, non-pathogenic in mice), and E-Wigeon/158 (wild bird origin H6N2, non-pathogenic in mice) were used as controls. The polymerase activity assays were performed in triplicate.

Viral RNP polymerase activity is a key determinant of influenza pathogenicity in mammalian hosts. NJ2501 and CZ56 were selected to perform a mini-genome luciferase reported assay. Two genotype H9N2 viruses with contrasting mouse virulence, A/chicken/Jiangxi/198/2019(H9N2) (JX198, highly pathogenic in mice) and XZ508 (non-pathogenic in mice) [28], as well as a wild bird origin H6N2 isolate A/Eurasian wigeon/Jiangxi/2018WB0158 (E-Wigeon/158, non-pathogenic in mice), were included as reference controls. As shown in Figure 7, JX198 exhibited the highest relative polymerase activity among all test viruses. The polymerase activity of NJ2501 was significantly higher than that of CZ56. Both NJ2501 and CZ56 displayed higher polymerase activity than the wild bird origin H6N2 isolate E-Wigeon/158. Notably, NJ2501 showed comparable polymerase activity to XZ508, while CZ56 possessed significantly lower polymerase activity than XZ508.

### Pathogenicity of the novel H6 isolates in chickens

**Figure 8.**
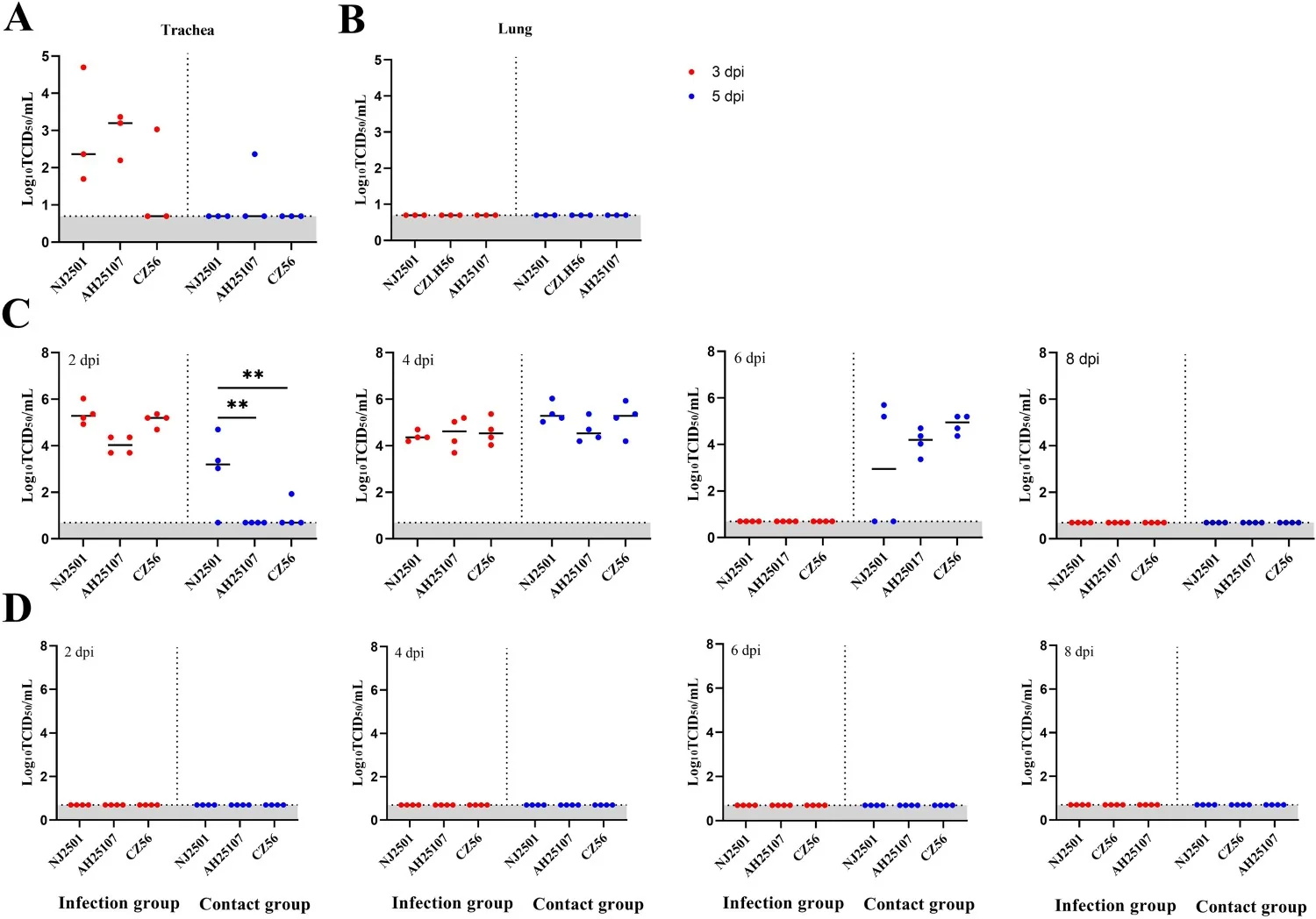
Replication and transmission of the H6 isolates in chickens. Groups of 10-day-old chickens were intranasally infected with 10^6^ TCID_50_ of each H6 isolate, and four naive chickens were introduced as a direct-contact group. At 3 and 5 dpi, three chickens from each infected group were euthanized, and trachea (A) and lung (B) samples were collected for viral determination. At 2, 4, 6, and 8 dpi, oropharyngeal (C) and cloaca (D) swabs from each group were collected, and viral titres were determined in MDCK cells.

To evaluate the pathogenicity of the H6 isolates in poultry, 10-day-old SPF chickens were intranasally infected with 10^6^ TCID_50_ of NJ2501, AH25107 and CZ56, respectively. Uninfected chickens were introduced as the direct-contact group. Infected chickens developed obvious clinical symptoms starting at 4 dpi (fur ruffling and open-mouth breathing), then began to recover at 6 dpi. At 3 and 5 dpi, three chickens per infection group were sacrificed, and trachea and lung samples were collected for viral detection. At 3 dpi, high viral titres were detected in the trachea of chickens infected with NJ2501 and AH25107, while only one of the chickens infected with CZ56 had detectable viral titre in trachea samples (Figure 8(A, B)). At 5 dpi, one chicken of AH25107 had detectable viral titres in trachea, while no virus was detected in any other trachea samples in other groups. Importantly, no viral titres were detected in the lungs of any groups at either 3 or 5 dpi. To further examine the viral shedding and transmission, oropharyngeal and cloacal swabs were collected from both infected and contact chickens at 2, 4, 6, and 8 dpi and titrated for virus. All infected chickens had viral shedding in oropharyngeal swabs at 2 and 4 dpi, and viral shedding ceased by 6 dpi. Among the contact chickens, three chickens in the NJ2501 contact group exhibited detectable viral shedding in oropharyngeal swabs at 2 dpi, whereas no shedding was observed in the other two contact groups. At 4 dpi, all contact chickens showed viral shedding with similar viral titres. By 6 dpi, only 2 chickens in the NJ2501 group were still shedding virus, while viral shedding persisted in the remaining contact group. At 8 dpi, no viral shedding was detected in oropharyngeal swabs of contact chickens. Notably, no viral shedding was detected in cloacal swabs from any inoculated or contact chickens throughout the entire experimental period. All these demonstrate that these novel H6 isolates can efficiently cause respiratory symptoms and transmission in chickens.

## The antigenicity of the novel H6 isolates

To assess whether antigenic variations existed among the novel H6 isolates, serum samples were collected from the remaining chickens at 21 dpi and subjected to cross-HI assays. The results showed the antiserum exhibited robust HI titres against the corresponding virus as well as the other two H6 viruses, with HI titres ranging from 1:320 to 1:1280 (Table 3). These results indicated that no substantial antigenic drift occurred among the H6 isolates.

**Table 3.** The antigenicity of the novel H6 isolates.

Virus	Antisera cross-HI titres		
	NJ2501	AH25107	CZ56
NJ2501	1:640	1:640	1:640
CZ56	1:1280	1:640	1:320
AH25107	1:640	1:640	1:640

## Discussion

The continuous emergence of the novel reassortant AIVs poses a great threat to both poultry industry and public health. In this study, we identified and characterized six novel reassortant H6N1 and H6N2 AIVs circulating in chickens with respiratory symptoms, all of which harbour internal genes from the G57 genotype H9N2 virus. Our findings provide further evidence that H9N2 viruses serve as a critical genetic backbone facilitating the generation of novel reassortant AIVs with enhanced fitness in poultry and increase zoonotic potential.

One of the most notable features of the H6 isolates described here is their conserved internal gene constellation originating from G57 H9N2 viruses. Since its emergence, G57 genotype has become the dominant H9N2 lineage in Chinese poultry [38–40] and has contributed internal genes to multiple zoonotic AIVs, including H7N9 [4], H10N8 [6], H5N6 [8], H10N3 [41] and H3N8 [42] viruses. These repeated involvement of G57 internal genes in novel reassortant viruses highlights their role in enhancing viral replication efficiency, host adaptability, and transmission [32, 39, 43, 44]. In line with this observation, these novel H6 viruses characterized in this study replicated efficiently in both avian and mammalian cells, and exhibited high pathogenicity in mice. These findings suggest that these novel H6 viruses possess the potential to cause cross-host infections in mammals and even humans.

Interestingly, previous studies have proposed that geese may represent an evolutionary “end-point” for H6 subtype AIVs, suggesting limited onward transmission or further reassortant once established in this host [45]. However, our surveillance data revealed that the genes of these six H6 isolates characterized in this study were derived from goose-origin H6 viruses (Figure 1(A)), yet these viruses are actively circulating in chickens and have undergone reassortment with H9N2 internal genes (Supplementary Figures 1 and 2). This finding challenges the notion that geese serve as a terminal host in the evolutionary trajectory of H6 viruses. Instead, our results suggest that goose-origin H6 viruses can be reintroduced into chickens and continue to evolve through genetic reassortment. Such dynamic interspecies transmission highlights a more complex ecological network than previously appreciated, in which geese may act not only as reservoirs but also as sources for the generation of novel reassortant viruses with enhanced adaptability. These observations underscore the importance of continued surveillance across multiple avian species to better understand the evolutionary pathways and cross-species transmission dynamics of H6 AIVs.

Receptor-binding specificity is a key determinant of host tropism and cross-species transmission [40]. In this study, we observed that wild type H6N2 isolate NJ2501 exhibited dual receptor-binding properties. Such dual specificity has been considered a hallmark of zoonotic potential in AIVs and has been reported in several human-infecting strains. Unexpectedly, none of the five wild type H6N1 isolates produced detectable binding to avian- or human-type receptors under the experimental conditions used, despite their robust replication in mammalian cells (Figure 5) and high virulence in mice (Figure 6). It should be emphasized that glycan binding assays for influenza viruses are affected by number confounding experimental variables. The glycopolymer probes format (Neu5Acα2-3Galb1-4GlcNAcb-sp3-PAA-biotin and Neu5Acα2-6Galb1-4GlcNAcb-sp3-PAA-biotin) in this study represent only one of many types of glycan-conjugated substrates [42, 46], although they have been widely utilized in influenza receptor-tropism studies. Other representative glycan reagents, even bearing identical sialic acid glycan sequences but differing in valency, space chemistry, carrier scaffold, or immobilization strategy, may produce distinct binding outcomes for the same virus. In this study, the H6N2 isolate NJ2501 exhibited dual receptor binding signals, whereas none of the H6N1 viruses showed detectable binding under the same experimental conditions. To resolve this apparent contradiction, we generated the reassortant virus rCZ56 by introducing the HA of CZ56 into the G57 genotype H9N2 (XZ508) backbone. Notably, rCZ56 displayed clear dual receptor binding affinity for both avian-like and human-like receptors, demonstrating that the HA protein of H6N1 intrinsically harbours dual receptor binding potential. This phenomenon may be attributable to multiple factors including relatively low binding affinity of the HA protein, high NA activity, or limitations of the *in vitro* assay system. Importantly, comparative sequence analysis revealed that key amino acid residues and structural regions within HA receptor-binding site were highly conserved among all six H6 isolates (data not shown). Together with their HA activity towards multiple species RBCs and efficient replication in both avian and mammalian cells (Figure 5), this indicates that these H6 isolates are capable of infecting and propagating in human respiratory epithelial cells. Collectively, our findings suggest that the receptor-binding properties of these H6 viruses may be more complex than indicated by *in vitro* assay alone and highlight their potential for adaptation to mammalian hosts.

The *in vivo* study further demonstrated that these H6 isolates enhanced pathogenicity in mammals. Notably, NJ2501 caused rapid weight loss and 100% mortality in mice without prior adaptation, highlighting its high virulence. In contrast, AH25107 and CZ56 exhibited comparatively lower pathogenicity, suggesting heterogeneity in virulence among these H6 isolates. Interestingly, NJ2501 showed markedly higher viral replication in the nasal turbinates than AH25107 and CZ56, indicating enhanced replication efficiency in the upper airway. Replication in the upper respiratory tract has been reported to facilitate viral shedding and transmission [33, 47, 48] and, in some cases, may contribute to increased pathogenicity by enabling efficient initial infection and rapid viral amplification [49]. Therefore, the enhanced replication of NJ2501 in nasal turbinates may be associated with its increased virulence observed in mice. To explore the molecular basis underlying such virulence divergence, we compared the RNP polymerase activity between NJ2501 and CZ56. The results revealed that both H6 isolates possessed markedly higher polymerase activity than the non-pathogenic wild bird origin H6N2 isolate E-Wigeon/158, highlighting the fitness gain conferred the G57 genotype H9N2 internal genes. Importantly, NJ2501 displayed significantly higher polymerase activity than CZ56, which may partially explain its increased virulence. Interesting, XZ508 exhibited polymerase activity comparable to NJ2501, yet XZ508 is non-pathogenic in mice [28]. This strongly indicates that the polymerase activity alone is insufficient to confer high pathogenicity in mice, and additional viral mechanisms are required to establish high virulence. Of note, the H6N2 and H6N1 isolates differ in their NA gene origin, NJ2501 carries an N2 segment derived from G57 genotype H9N2 virus, whereas CZ56 and AH 25017 carry N1 segments from H5N1 viruses. Such a difference in NA genetic background may alter virus host interactions and further modulate virulence in mammals. Collectively, the differential pathogenicity observed in mice is likely a multifactorial outcome driven by the combinatorial effects of multiple viral gene segments rather than a single molecular determinant. Therefore, the molecular mechanisms responsible for the varied mouse virulence among these novel H6 reassortants require further investigation.

In addition to their mammalian pathogenicity, these H6 viruses demonstrated efficient infection and transmission in chickens. Viral shedding was detected in both infected and contact chickens (Figure 8), indicating effective direct-contact transmission of these novel H6 viruses. The ability to spread efficiently within poultry is a critical factor for viral maintenance and amplification, which increases the likelihood of further reassortment and spill-over events. Compared with previous reports in which H6 viruses showed limited transmission in poultry [26], the strains described here appear to have acquired enhanced transmissibility, possibly due to their adapted internal genes of H9N2 virus origin.

One limitation of the present study is the absence of authentic non-reassortant parental H6 viruses as direct experimental control, which restricts our ability to precisely quantify gene-segment specific contribution to viral phenotypes. Future reverse-genetics studies will be required to dissect the functional role of individual segments originating from H9N2 and H5N1 donors.

In conclusion, our study highlights the evolution of H6 AIVs through reassortment with G57 genotype H9N2 viruses and demonstrates that such reassortment can result in AIVs with enhanced pathogenicity and cross-species transmission potential. These findings underscore the importance of continuous surveillance of AIVs in poultry flocks in China, particularly those carrying H9N2 internal genes, as they may serve as precursors to zoonotic or even pandemic AIVs.

## Supplementary Material

SFigure 1.jpg

SFigure 2.jpg
